# Clinical features and prognosis of paediatric rhabdomyosarcoma with bone marrow metastasis: a single Centre experiences in China

**DOI:** 10.1186/s12887-021-02904-9

**Published:** 2021-10-21

**Authors:** Cheng Huang, Binglin Jian, Yan Su, Na Xu, Tong Yu, Lejian He, Xue Zhang, Yi Liu, Mei Jin, Xiaoli Ma

**Affiliations:** 1 Medical Oncology Department, Pediatric Oncology Center, Beijing Children’s Hospital, Capital Medical University, National Center for Children’s Health, Beijing Key Laboratory of Pediatric Hematology Ocology, Key Laboratory of Major Diseases in Children, Ministry of Education, Beijing, China 100045; 2 Imaging Center, Beijing Children’s Hospital, Capital Medical University, National Center for Children’s Health, Beijing, China 100045; 3 Department of Pathology, Beijing Children’s Hospital, Capital Medical University, National Center for Children’s Health, Beijing, China 100045; 4 Beijing Key Laboratory of Pediatric Hematology Oncology; National Key Discipline of Pediatrics (Capital Medical University); Key Laboratory of Major Diseases in Children, Ministry of Education; Hematology Oncology Center, Beijing Children’s Hospital, Capital Medical University, National Center for Children’s Health, Beijing, China 100045

**Keywords:** Rhabdomyosarcoma, Bone marrow metastasis, PAX-FOXO1 fusion genes, Paediatric, China

## Abstract

**Background:**

The aim of this study was to summarize the clinical characteristics, therapeutic effects and prognosis of patients with rhabdomyosarcoma (RMS) and bone marrow metastasis, improve the understanding of this disease.

**Method:**

This was a single-institution retrospective study involving the children with RMS, who presented with bone marrow metastasis at initial presentation to our hospital between 1st, Jan, 2006 and 31st, Dec,2019. Follow-up concluded on 31st, Dec, 2020 and the clinical data were collected and analysed.

**Result:**

Between 1st Jan 2006 and 31st Dec 2019, 13 eligible patients presented to our hospital, including 10 males and 3 females, these eligible patients accounted for 4.5% of all RMS patients. The median age at onset was 5.6 years (range 1.7-14 years). The patients not only had unfavourable primary sites, but also had multiple metastases. The bone marrow aspirate samples of the patients comprised 8-95% blast-like cells. Nine of 13 patients were misdiagnosed with haematological malignancies or other solid tumours. With respect to histology, four of 13 children were classified as embryonal RMS and nine as alveolar RMS. Eleven patients underwent PAX-FOXO1 fusion testing; eight had the POX- FOXO1 fusion gene. Immunohistochemically(IHC) analysis revealed that the tumour cells were positive for Desmin, Vimentin, Myo-D1 and Myogenin. More importantly, the patients had extremely poor prognoses, the median EFS was 12.0 months (range 3-28.3 months) and the median OS was 27.0 months (range6-46.2 months).

**Conclusion:**

This study demonstrates that children with RMS and bone marrow metastasis usually exhibit atypical primary sites and multiple metastases, with presentation mimicking haematological malignancies or other solid tumors at initial presentation. Pathology and IHC analysis combined with POX-FOXO1 fusion gene detections can effectively confirm the diagnosis. These patients are more likely to relapse or progress during early treatment and are prone to intracranial metastasis. While multidisciplinary therapy combined with Temozolomide may prevent it, further prospective research is required to evaluate the therapeutic effects.

## Background

Rhabdomyosarcoma (RMS) is the most common soft-tissue sarcoma in children and adolescents [1]. Although children with localized RMS have a relatively favourable prognosis, more than 35% of children presented with metastasis at initial presentation [2]. The lymph nodes and lungs are the most common sites of RMS metastasis. In contrast, bone marrow metastasis is uncommon, occurring in only 6% of all RMS cases [3]. Due to the atypical clinical features and bone marrow pathology, most patients are misdiagnosed with haematological malignancies or other solid tumours at initial diagnosis presentation, and these results in delayed treatment [4]. More notably, those patients often present with highly aggressive disease and poor prognosis, they either do not respond to conventional therapies or relapse after treatment [5]. To date, there are few published reports on the clinical characteristics and treatment outcomes of these children. Therefore, in this article, we analysed the clinical characteristics, therapeutic efficacy and prognosis of children with RMS involving bone marrow metastases and explored the therapeutic schedule for this disease. The ultimate goal of this study was to contribute to improve prognosis for these patients.

## Material and methods

### Patients

This single-institution retrospective analysis included patients with newly diagnosed RMS who had bone marrow metastasis according to bone marrow pathology and were aged less than 18 years at diagnosis. The study was conducted at our centre between 1st, Jan, 2006 and 31st, Dec, 2019. This trial was approved by the Ethics Committee of Beijing Children’s Hospital (2018-k-106). Informed consent was obtained from all parents or guardians of the participating children.

### Stageing

All patients were stratified into risk groups according to the Intergroup Rhabdomyosarcoma Study (IRS) staging system and theTNM pre-treatment staging system (According to this system, staging is determined by the primary tumour site, degree of tumour invasion, nodal status, and the presence or absence of metastases; staging is performed solely based on the preoperative workup of imaging and physical exam) [1, 6]. The two staging measures were combined to guide the patient’s therapy.

### Treatment protocol

Patients received multidisciplinary treatment, which comprised chemotherapy, surgery and radiotherapy (RT). Children whose tumours could be removed, received surgery firstly; for the children whose tumours could not be surgically removed, received biopsy and surgery was planned after 4 -8 courses of chemotherapy. Children with IRS III-IV were required to undergo RT. As patients with alveolar RMS(ARMS) are more likely to relapse, those classified in the IRS I-II were also required to undergo RT. With respect to chemotherapy, before 2016, children in the low-risk group received theVAC/VA (V: Vincristine, A: Actinomycin, C: Cyclophosphamide) chemotherapy regimen for 8-10 cycles, Patients in the median-risk group received the VAC/VTC (T:Topotecan) chemotherapy regimen for 14-16 cycles and patients in the high-risk group received VAC/VDC/IE (D:Doxorubicin, I: Ifosfamide, E:Etoposide) regimen for 14-16 cycles. After 2016, patients were treated in accordance with the Chinese Children Cancer Group-Rhabdomyosarcoma-2016 (CCCG-RMS-2016) protocol, which is based on the IRSG protocol and EpSSG protocol; Carboplatin and Iosfamide or other centrally penetrating chemotherapy drugs were added to the regimen for patients with central nervous involvement [7]. All patients received bone marrow aspiration or biopsy to identify the presence of bone marrow infiltration. Moreover, in order to prevent central recurrence, Temozolomide 150 mg/m2 was taken orally every 4 weeks for a total of eight times.

### Statistical analysis

The study follow-up ended on 31st, Dec,2020. The Kaplan-Meier method was used to estimate the distribution of EFS (event-free survival) and OS (overall survival) and the patient groups were compared with the log-rank test. EFS were defined as the time from the beginning of therapy to the first disease progression, recurrence, or death from any cause. OS was defined as the time from therapy initiation to death from any cause or conclusion of the study. Data analysis was performed using SPSS version 22.0.

## Results

### Clinical characteristics of the patients

A total of 291 children were diagnosed with RMS at our centre from 1st, Jan,2006 to 31st, Dec, 2019, but only 13 (4.5%) children had bone marrow metastasis; of these,10 were male and 3 were female with a median age was 5.6 years (range 1.7-14 years). The median courses of the disease was 2 months (range 0.3-4 months). The primary sites were almost all located in unfavourable sites, and were accompanied by multiple metastatic tumours (range 3-17);11/13 had bone involvement. The bone marrow aspirate samples of the patients comprised 8-95% blast-like cells. The median tumour size was 6.6 cm (range 2.4-12.9 cm). Intriguingly, several patients presented with fever, joint pain, abdominal distension or other atypical symptoms, the lower peripheral haemogram results and similar bone marrow cytology resulted in 9 of the 13 children being misdiagnosed with haematological malignancies or other solid tumours at initial diagnosis. The median delay in diagnosis was 1 month (range 0.5-3 months). Histologically, the majority of patients (9 /13) were classified as ARMS while 4/13 cases were classified as embryonic RMS(ERMS). Eleven children underwent special PAX-FOXO1 fusion gene testing,8 of the 13 children harboured the PAX-FOXO1 gene fusion. The immunohistochemical (IHC) results revealed that the patients were positive for the myogenic biomarkers Vimentin, Myo-D1, Myogenin and Desmin. The characteristics of the 13 eligible patients are presented in Table 1.

**Table 1:** Clinical characteristics of the 13 patients with RMS and bone marrow metastasis

Case	Sex	age (year)	Primary site	Initial diagnosis	A A	Size (cm)	Oberlin Score	Total	Bone	PAX/ FOXO	Pathol- ogical	Immunohistochemically
								metastasis	metastasis			
**1**	M	11.8	Groin	NHL	N	9	4	7	P	N	ERMS	Desmin(+),Vimentin(+), MyoD1(+), Myogenin(+)
**2**	M	3.2	Chest wall	PPB	N	5	4	8	P	–	ERMS	Desmin(+), MyoD1(+), Myogenin(+)
**3**	F	2.9	chest wall	AL	Y	7.7	4	7	P	P	ARMS	Desmin(+),Myogenin(+), Vimentin(+),
**4**	M	2	Pelvic cavity	AML	Y	4	4	7	N	–	ARMS	Desmin(+), Myoglobin(+)
**5**	M	1.7	Lower jaw	AML	N	3.7	4	17	P	P	ARMS	Desmin(+),Vimentin (+), Myogenin(+)
**6**	M	5.8	cranial base	PPB	Y	6.6	4	4	P	N	ERMS	Desmin(+), MyoD1(+),Myogenin(+)
**7**	M	12	Nasopharynx	RMS	N	4.5	4	10	P	P	ARMS	Desmin(+), MyoD1(+), Myogenin(+)
**8**	M	8.4	Palm	NB	Y	3.1	4	9	P	P	ARMS	Desmin(+), MyoD2(+), Myogenin(+)
**9**	M	5.6	Bladder	RMS	N	7	4	4	P	N	ERMS	Desmin(+), MyoD1(+),Myogenin(+)
**10**	F	13	Mediastinum	PPB	Y	8.4	4	3	N	P	ARMS	Desmin(+), Vimentin(+), Myogenin(+)
**11**	F	14	Sole	RMS	N	2.4	4	4	P	P	ARMS	Desmin (+), Vimentin(+), MyoD1(+), Myogenin(+)
**12**	M	5.4	Pelvic cavity	HL	N	10	4	9	P	P	ARMS	Desmin(+), MyoD1(+), Myogenin(+)
**13**	M	2.1	Pelvic cavity	RMS	N	12.9	4	6	P	P	ARMS	Desmin(+), MyoD1(+),Myogenin(+)

*Abbreviations*: *M* Male, *F* Female, *NB* Neuroblastoma, *NHL* Non-Hodgkin lymphoma, *PPB* Pleuropulmonary blastoma, *AL* Acute leukemia, *AML* acute myelocytic leukemia, *HL* Hodgkin's Lymphoma, *AA* Aplastic anemia, *ARMS* Alveolar rhabdomyosarcoma, *ERMS* Embryonal rhabdomyosarcoma, *N* NO, *Y* Yes, *P* PosItive, *N* Negative

### Therapeutic effects

**Fig. 1:**
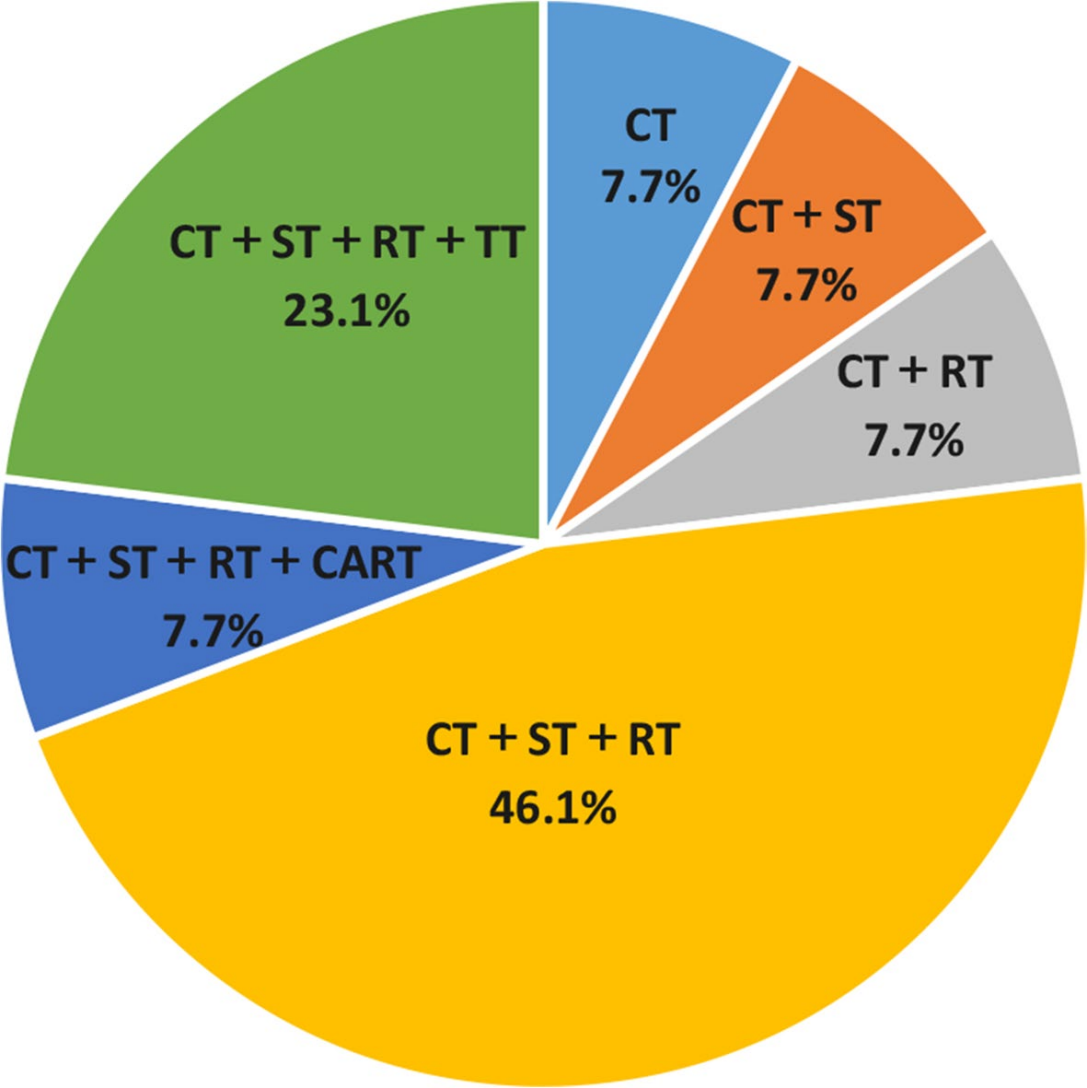
The overall treatment status of the 13 children. Abbreviations: CT: chemotherapy; RT: radiotherapy;ST: Surgery treatment; CAR-T: Chimeric Antigen Receptor T-Cell Immunotherapy; TT: Targeted treatment

All patients received chemotherapy;11/13 patients received RT, 11 /13 patients accepted surgery and 8/13 patients took Temozolomide orally. Before 2016, 3/ 5 children had experienced local and distant progression during early treatment, they did not undergo secondary surgery, and all died due to the intracranial metastases. Notably,2 /5 children had relapsed after treatment. One patient had lung metastasis after 2 months of withdrawal chemotherapy; he was treated with the chemotherapy, CAR-T (Chimeric Antigen Receptor T-Cell Immunotherapy). Unfortunately, head CT showed extensive intracranial metastasis. Despite receiving RT and surgery, he eventually died due to progression of the intracranial metastasis. Among the remaining eight children (after 2016), two had intracranial metastasis at initial visit, they developed inoperable tumors during early chemotherapy (4 months later) and died during the RT and chemotherapy. The remaining six children not only received chemotherapy, surgery and RT, and also took Temozolomide orally to prevent intracranial metastasis. Among these six children, one dead as the RT and chemotherapy could not control tumour progression. Among the five surviving children, four children experienced local or metastatic recurrence, received second chemotherapy, three patients underwent surgery for a second time and one patient with mutual-bone metastasis received RT and target treatment(Temsirolimus) (Table 2, Fig. 1).

**Table 2:** Therapeutic schedule and outcomes of the 13 patients with RMS and bone marrow metastasis

Case	Treatment	Take	From onset	Main site of	Treatment	Follow-up	Outcome
		Temozolomide	to progression(m)	Progression	after relapse	(m)	
1	CT	NO	3	Primate site, lung, intracalvarium	CT	6	death
2	CT + ST	NO	6	Primate site,lung,intracalvarium	CT	8	death
3	CT + RT + ST	NO	6	Intracalvarium	CT + TT	8	death
4	CT + RT + ST	NO	15	Primary site, Lymph node	CT	27	death
5	CT + RT + ST	NO	13.5	Intracalvarium	CT + ST + RT + CART	36.5	death
6	CT + RT	Yes	4.3	Intracalvarium	CT	8	death
7	CT + RT + ST	Yes	4.8	Lung, bone marrow	CT	16	death
8	CT + RT + ST	Yes	9	Bone marrow, Intracalvarium	CT + ST + RT + TT	12.3	death
9	CT + RT + ST	Yes	19	Bone	CT + RT	21	Survive
10	CT + RT + ST	Yes	21.2	Primary site, lymph nodes	CT + ST + RT	27.2	survive
11	CT + RT + ST	Yes	12	Lymph nodes	CT + ST + RT	27. 6	survive
12	CT + RT + ST	Yes	28.3	Primary site	CT + ST + RT + TT	46.2	survive
13	CT + RT + ST	Yes	/	/	/	14.5	survive

*Abbreviations*: *CT* Chemotherapy, *RT* Radiotherapy, *ST* Surgery treatment, *CAR-T* Chimeric Antigen Receptor T-Cell Immunotherapy, *TT* Targeted treatment

### Survival

**Fig. 2:**
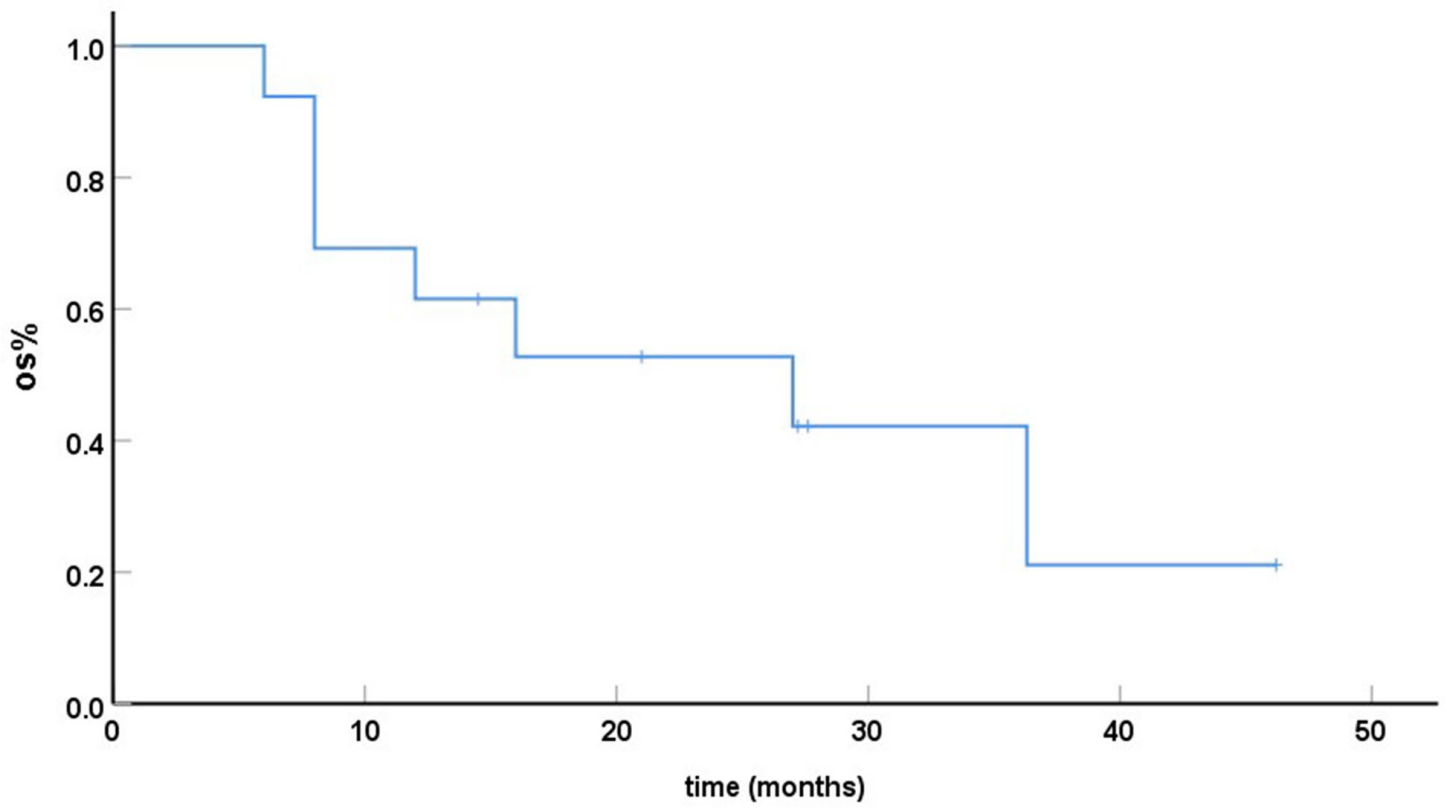
The overall survival time of 13 children with RMS and bone marrow metastasis

As of 31st Dec, 2020, 12/13 patients had relapsed or experienced disease progression and 8/13 had died. All deaths were related to the progression or recurrence of primary tumours and metastases. The median EFS time was12.0 months (range 3-28.3 months) and the median OS time was 27 months (range 6-46.2 months). The Kaplan Meier estimates of OSare showed on Fig. 2.

## Discussion

RMS is an aggressive sarcoma that accounts for 50% of all soft tissue sarcoma in children and adolescents. It is characterized by highly malignancy and short courses. In particular, among children with bone marrow metastasis, the course of the disease can be less than 1 - 2 months [8, 9]. Due to its similar symptoms and indistinguishable histopathological results, RMS with bone marrow metastasis may mimic haematological malignancies [10–12]. There are several case reports of ARMS, accompanied by extensive lymph node metastasis, with naive and atypical bone marrow infiltrating cells in the bone marrow cytology smear and without an identifiable primary tumour, these cases are often confused with acute leukemia or lymphoma and, thus, presented a diagnostic problem [13, 14].

In the current study, all children presented with multiple metastases (such as the lymph nodes, lung, bones, etc.) at the time of initial diagnosis. The median course of the disease was only 2 months; four cases survived for less than 1 month. Due to the extensive metastases and unclear primary sites together with juvenile or rare cells in the bone marrow smears, five children were misdiagnosed with Leukemia or NHL and four were misdiagnosed with other solid tumours. Research has shown that the expression of skeletal muscle differentiation markers has high specificity and sensitivity and important value in primary and metastasis of RMS, these markers are often used as markers for RMS diagnosis [15]. Therefore, in consultation with the pathology departments of three hospitals, histological examination was performed and Desmin, Myogenin, MyoD1 and other muscle-specific actins in immunohistochemical stains were detected. Moreover, the PAX-FOXO1 fusion genes were detected. The results of these investigations eventually confirmed that the patients had RMS. The majority of patients (9 /13) were diagnosed with ARMS. Of the nine cases of ARMS, eight cases were PAX-FOXO1 gene-positive.

The PAX-FOXO1 fusion genes, as a transcriptional activator, affect multiple oncogenic pathways. Ommer J [16] et al. confirmed that antisense-mediated loss of PAX-FOXO1 results in cell death, underscoring the addiction of FP-RMS cells to the fusion protein. ARMS is characterized by the presence of balanced reciprocal translocations and the PAX-FOXO1 fusion genes is expressed in many cases, making it a clear diagnostic aid to confirm an ARMS diagnosis in many pathology laboratories worldwide [17, 18]. Recent studies have suggested that about 80% of ARMS cases have either t(1;13)(p36;q14) or t(2;13)(q35;q14), each of which results in the formation of the fusion oncogenes PAX -FOXO1, respectively [19]. Research also suggests that children with the PAX-FOXO1 fusion gene tend to have metastatic tumours.

In the current study, eight ARMS patients had the PAX-FOXO1 fusion gene, accompanied by multiple metastases, most of these patients had a dismal prognosis, with recurrence occurring during treatment. Additionally, the primary sites in these patients were usually located in unfavorable sites (such as the pelvic cavity, mediastinum, or limbs). Moreover, the Oberlin score was usually higher than three, there was commonly bone marrow suppression, and patients were more likely to experience complications such as anaemia/bleeding and infection during treatment, which also confer poorer prognosis. Therefore, RMS with bone marrow metastasis may be a fatal diagnosis and new therapies are required to relieve the disease [20].

Despite the combination of chemotherapy, RT and surgery to improve the clinical outcomes, the survival of children with metastatic RMS remains poor, with three-year OS ranging between 34 and 56% [21, 22]. The identification of novel treatments has become a major focus of research on RMS. The COG randomized clinical trial of 68 children with recurrent RMS showed that children with RMS who received Temsirolimus had superior EFS over those who received Bevacizumab after relapse [23]. In the current study, two children received Bevacizumab after relapse; however, these children only survived for 2 to 4 months. One child received Temsulolimus with chemotherapy after relapse and was still surviving at the conclusion of the study, with an OS of 46.2 months.

More importantly, among RMS metastatic patients, fatality mainly occurs in the presence of intracranial metastases [24]. Interestingly, the current study showed that children with bone marrow metastases were more inclined to intracranial metastases, four of five children enrolled in the study before 2016 inclined to central system metastasis and all died. The oral bioavailability of Temozolomide is nearly 100% and it easily penetrates the blood-brain barrier; thus, it is used to treat central nervous system tumours [25, 26]. The European Paediatric Soft-tissue Sarcoma Study Group Phase II trial also found that Temozolomide combined with VI improved the chemotherapy efficacy for patients with relapsed or refractory RMS [27]. Therefore, Temozolomide combined with chemotherapy and RT was adopted to prevent brain metastasis in the next eight cases of the current study. While two of the eight children had extensive intracranial metastasis at initial presentation progress, the remaining six patients had no evidence of intracranial metastasis throughout the study follow-up.

## Conclusions

In conclusion, this study indicates that RMS with bone marrow metastasis is usually accompanied by multiple metastases and is difficult to distinguish from haematological malignancies and other solid tumours. Furthermore, those patients are more likely to progress and relapse during early treatment and tend to have intracranial metastasis. Multimodal treatment combined with Temozolomide may be an option to prevent the intracranial metastasis. However, given the small sample sizes in the current study, we are currently undertaking prospective research to further evaluate the clinical value of Temozolomide for RMS children.

## Data Availability

The data used and analyzed during the current study are available from the corresponding author on reasonable request.

## Abbreviations

RMS: Rhabdomyosarcoma
ARMS: Alveolar RMS
RT: radiotheraphy
V: Vincristine
A: ctinomycin
C: Cyclophosphamide
T: Topotecan
D: Doxorubicin
I: Ifosfamide
E: Etoposide
EFS: Event-free survival
OS: Overall survival
ERMS: Embryonic RMS
CAR-T: Chimeric Antigen Receptor T-Cell Immunotherapy
